# Current Diagnostic Pathways for Alzheimer’s Disease: A Cross-Sectional Real-World Study Across Six Countries

**DOI:** 10.3233/ADR230007

**Published:** 2023-06-29

**Authors:** Sophie Roth, Nerida Burnie, Ivonne Suridjan, Jessie T. Yan, Margherita Carboni

**Affiliations:** a Roche Diagnostics International Ltd, Rotkreuz, Switzerland; b General Practice, South West London CCG, London, UK; c Roche Diagnostics, Santa Clara, CA, USA

**Keywords:** Alzheimer’s disease, cognitive dysfunction, dementia, diagnosis, neurology, neuropsychological tests, standard of care, surveys and questionnaires

## Abstract

**Background:**

Diagnostic pathways for patients presenting with cognitive complaints may vary across geographies.

**Objective:**

To describe diagnostic pathways of patients presenting with cognitive complaints across 6 countries.

**Methods:**

This real-world, cross-sectional study analyzed chart-extracted data from healthcare providers (HCPs) for 6,744 patients across China, France, Germany, Spain, UK, and the US.

**Results:**

Most common symptoms at presentation were cognitive (memory/amnestic; 89.86%), followed by physical/behavioral (87.13%). Clinical/cognitive tests were used in > 95%, with Mini-Mental State Examination being the most common cognitive test (79.0%). Blood tests for *APOE* ɛ4/other mutations, or to rule out treatable causes, were used in half of the patients. Clinical and cognitive tests were used at higher frequency at earlier visits, and amyloid PET/CSF biomarker testing at higher frequency at later visits. The latter were ordered at low rates even by specialists (across countries, 5.7% to 28.7% for amyloid PET and 5.0% to 27.3% for CSF testing). Approximately half the patients received a diagnosis (52.1% of which were Alzheimer’s disease [AD]). Factors that influenced risk of not receiving a diagnosis were HCP type (higher for primary care physicians versus specialists) and region (highest in China and Germany).

**Conclusion:**

These data highlight variability in AD diagnostic pathways across countries and provider types. About 45% of patients are referred/told to ‘watch and wait’. Improvements can be made in the use of amyloid PET and CSF testing. Efforts should focus on further defining biomarkers for those at risk for AD, and on dismantling barriers such low testing capacity and reimbursement challenges.

## INTRODUCTION

Global prevalence estimates for dementia range from 0.6– 40.8% in Europe (in people aged 60– 64 years and 90 and older, respectively [1]), to 5.6% in China (in those 65 years and older [2]), and 11.0% in the United States (US) [3]. Alzheimer’s disease (AD), the most common form of dementia, accounts for 60– 70% of cases worldwide [4]. The prevalence of AD was estimated as 22.5% in people aged 85 and older in Europe [5], and 17.9– 33.5% in people aged over 80 in China [6]. Prevalence is expected to increase globally [1,7], more than doubling from 6.07 million in 2020 to 13.85 million in 2060 in the US alone [7].

In 2019, AD and other forms of dementia were the seventh-highest cause of death globally with an estimate of 1,639,085 deaths [8]. Individuals with AD have a higher mortality rate than controls (hazard ratio [HR], 3.70 [9]). AD-related deaths in people aged ≥50 were found to have more than doubled in Europe from 1994 to 2013 [10]. In addition to increasing mortality, AD greatly reduces quality of life in sufferers as well as their caregivers and imposes a considerable societal and economic burden [11].

AD progresses in a continuum from preclinical disease (normal cognition with brain pathology) to a prodromal stage of mild cognitive impairment (MCI) and finally to clinically-measurable dementia [11]. It is challenging to distinguish the prodromal stage of MCI, or very early stages of AD, from the cognitive decline that accompanies normal aging [12]. Partly for this reason, missed or delayed diagnoses of dementia (regardless of cause) are thought to be highly prevalent in primary care [13], and over 60% of persons with dementia are thought to be undiagnosed globally [14].

Historically, there have been no treatment options for AD. However, a recent review identified 21 disease-modifying therapies (DMTs) for AD in phase III clinical trials and an additional 71 in phase II trials [15]. This intense development focus will herald a new era for AD therapeutics over the next decade, as indeed evidenced by the accelerated approval of lecanemab by the US FDA [16]. DMTs are likely to be more effective if offered earlier [17–20]. Therefore, timely and accurate identification of patients with AD has become all the more critical.

Available evidence suggests substantial heterogeneity in the pathways through which patients presenting with cognitive complaints or impairments are ultimately investigated for amyloid pathology [21–23], with various biomarkers, neuropsychiatric tests, and diagnostic criteria contributing to variability [12].

The aim of this cross-sectional study was to describe the diagnostic pathway for patients presenting with cognitive impairments or complaints, from first presentation to diagnosis or other outcomes (such as a referral to another physician or advice to ‘watch and wait’), across six countries. Focus areas were symptoms at presentation, assessments ordered and/or conducted, as well as number of appointments. Results were stratified by physician specialty and country.

## MATERIALS AND METHODS

This study [24] is based on 1) a comprehensive quantitative physician online survey, conducted by Hall and Partners (UK) on behalf of Roche Diagnostics International; 2) patient data extracted by the responding physicians into patient record forms (PRFs). Similar methods have been used in earlier studies [25–28].

### Physician survey

A total of 32,533 healthcare professionals (HCPs) in China, France, Germany, Spain, UK, and US were invited to participate through specialized fieldwork experts and online panels. HCPs included primary care physicians (PCPs), specialists (geriatricians, neurologists, or psychiatrists), and nurses (in the UK only). This large pool of potential participants was contacted in order to achieve the predefined enrolment target of 1,695 within a reasonable time, after which the survey was closed.

Participants needed to be familiar with aspects of AD and diagnostic biomarkers and involved in diagnosing subjective or objective cognitive complaints or impairments due to AD or other types of dementia. HCPs based in China, France, Germany, UK, and US needed to have access to patient health records, and US HCPs needed to be board-certified.

Each participant answered questions in a 20-min online survey and completed PRFs for consecutive patients presenting with cognitive impairments/complaints, beginning with their most recent patient seen in the last 3 months prior to the survey. The PRFs requested information on demographics, symptoms, presentation pathway, number of appointments, diagnostic actions taken, referrals, diagnosis, and time taken to reach an outcome.

No data were collected directly from patients. Fieldwork was completed between 10 October and 15 November 2021.

Insights from a qualitative HCP survey and accompanying advisory board meetings, conducted in parallel with the quantitative survey described here, are reported separately [Suridjan I, van der Flier WM, Monsch AU, Bernie N, Baldor R, Sabbagh M, Vilaseca J, Cai D, Carboni M, Lah JL; unpublished data].

### Data management and statistical analysis

Field data management was conducted according to standard operating procedures (Supplementary Table 1). Raw data were transferred into a study database, with data cleaning (e.g., using plausibility checks for data ranges). All data analyses and visualizations were conducted by a Roche data scientist using SAS Studio version 3.81 (SAS Institute, North Carolina, US), except the Sankey diagram, which was built using the SankeyMATIC online tool (https://sankeymatic.com/build/; accessed 22 March 2022).

**Table 1:** Summary of patients presenting with cognitive impairments/complaints by type of consultation, initiator, and visits, by country

	All n (%)	US n (%)	China n (%)	UK n (%)	France n (%)	Germany n (%)	Spain n (%)
**All**	6,744 (100.0)	1,738 (100.0)	1,204 (100.0)	1,056 (100.0)	871 (100.0)	852 (100.0)	1,023 (100.0)
**First consultation for symptoms of cognitive impairment/cognitive complaints:**							
Yes	2,836 (42.1)	725 (41.7)	599 (49.8)	397 (37.6)	393 (45.1)	351 (41.2)	371 (36.3)
No	2,941 (43.6)	796 (45.8)	525 (43.6)	341 (32.3)	341 (39.2)	403 (47.3)	535 (52.3)
Missing/unknown	967 (14.3)	217 (12.5)	80 (6.6)	318 (30.1)	137 (15.7)	98 (11.5)	117 (11.4)
**Initiation of interaction for symptoms of cognitive impairment/cognitive complaint by:**							
Survey physician	1,998 (29.6)	499 (28.7)	442 (36.7)	229 (21.7)	297 (34.1)	248 (29.1)	283 (27.7)
Referral from another physician	794 (11.8)	178 (10.2)	32 (2.7)	300 (28.4)	117 (13.4)	69 (8.1)	98 (9.6)
Patient/family	3,906 (57.9)	1,056 (60.8)	708 (58.8)	522 (49.4)	453 (52.0)	529 (62.1)	638 (62.4)
Missing/unknown	46 (0.7)	5 (0.3)	22 (1.8)	5 (0.5)	4 (0.5)	6 (0.7)	4 (0.4)
Number of visits to investigate symptoms of cognitive impairment/cognitive complaints *							
Mean	2.81	2.88	2.6	2.16	2.62	2.91	3.72
Median	2	2	2	2	2	2	3
Min	1	1	1	1	1	1	1
Max**	85	60	36	45	30	80	85

UK, United Kingdom; US, United States of America * Data are absolute number of visits ** A small fraction of the population (3.8% [258/6,744]) had > 7 visits; 59% (152/258) of whom were patients under long-term management by the HCP for “general health or another comorbidity”, who had presented with a cognitive complaint.

Study results and SAS programming were quality-checked. After analysis by the assigned study statistician, a second quantitative scientist reviewed and checked the study results along with the corresponding programming codes used to generate the results.

Descriptive analyses included number (%) for categorical variables and mean (median, min, max) for continuous variables. Due to the small sample size of each individual specialty, they were pooled into an overall “specialist” group for analyses. No statistical tests were conducted. An absolute difference of more than 10% between categories was generally considered worthy of detailed description, e.g., for differences across countries or settings, although this was not strictly followed. Kaplan-Meier survival curves and Cox proportional-hazards regression models were conducted to estimate time to final diagnosis and risk of not receiving a final diagnosis. In the Cox analysis, we adjusted for the following covariates simultaneously: physician specialty type (PCP versus specialist), patient age group (<45 years, 45– 55 years, 56– 65 years, 66– 75 years, and≥76 years), country (China, UK, France, Germany, Spain, and US), patient symptoms at encounter (any physical/behavioral symptoms [such as getting lost in familiar locations], symptoms related to cognitive skills [such as difficulty with problem solving], symptoms related to language [such as struggling to find the right words], symptoms related to disorientation, and symptoms related to memory [such as difficulty with recognition of familiar people]) as well as source of initiation of interaction for symptoms of cognitive impairment (i.e., survey physician, referral from another physician, or patient or family).

### Ethical conduct

Consent was provided by participating HCPs. No data were collected directly from patients, and patient-related data provided by HCPs were anonymized, i.e., did not contain any identifying information. Hence, according to standard practice [26], no institutional review board/ethics committee approvals were necessary. Research complied with market research protocols, compliance requirements, data protection/privacy policies, and adverse event reporting guidelines. The physician survey complied with all industry regulations, including the Market Research Society, British Healthcare Business Intelligence Association, European Pharmaceutical Market Research Association, and General Data Protection Regulation guidelines. Professionals involved in the physician survey were fully trained in adverse event reporting by British Healthcare Business Intelligence Association and Roche Diagnostics International.

## RESULTS

### Healthcare provider characteristics

Of the 32,533 HCPs who received the survey invitation, 1,694 participated, completing 6,744 PRFs (three to seven PRFs per HCP; mean 3.98 [data not shown]); all patients had been seen in the 3 months prior to the survey. The number of HCPs ranged from 210 in Germany to 455 in the US (Supplementary Table 2). Overall, 38.8% were PCPs and 59.7% were specialists; nurses (*n* = 25) were recruited only in the UK. The proportion of PCPs was lowest in China (16.7%). Among specialists, the proportions of individual specialties were similar across most countries (geriatricians: 11.0% to 16.7%; neurologists: 19.6% to 23.8%; psychiatrists: 19.2% to 23.8%); exceptions were Germany, where the proportion of geriatricians was low (6.2%), and China, where the proportion of neurologists and psychiatrists was high (33.3% each). By practice type, academic/tertiary/regional hospital was the most common overall (34.4%), followed by office practice (32.0%). Spain had the highest proportion of academic/tertiary/regional hospitals (59.8%), UK the highest proportion of large general/regional hospitals (27.1%), and China the highest proportion of community/secondary hospitals (29.7%). China had no office practices.

**Table 2:** Summary of assessments used to investigate symptoms of cognitive impairment/cognitive complaints, by appointment

	Appointment				
	First n (%)	Second n (%)	Third n (%)	>Third n (%)	Any n (%)
All	6,662 (100.0)	4,663 (100.0)	2,512 (100.0)	1,236 (100.0)	6,662 (100.0)
Cognitive tests
Any cognitive tests	5,917 (88.8)	3,340 (71.6)	1,483 (59.0)	605 (48.9)	6364 (95.5)
Standard psychological/psychiatric evaluation	4,016 (60.3)	1,546 (33.2)	655 (26.1)	263 (21.3)	4,589 (68.9)
MMSE	4,284 (64.3)	1,888 (40.5)	647 (25.8)	261 (21.1)	5,262 (79.0)
Mini-Cog	1,860 (27.9)	909 (19.5)	370 (14.7)	160 (12.9)	2,606 (39.1)
ADAS-Cog	956 (14.4)	617 (13.2)	304 (12.1)	112 (9.1)	1,585 (23.8)
GPCOG	639 (9.6)	483 (10.4)	241 (9.6)	95 (7.7)	1,214 (18.2)
CANTAB mobile device test	278 (4.2)	287 (6.2)	175 (7.0)	83 (6.7)	672 (10.1)
Cognigram, Cognivue, Cognision or ANAM device test	316 (4.7)	347 (7.4)	215 (8.6)	97 (7.8)	784 (11.8)
BEHAVE-AD	578 (8.7)	480 (10.3)	261 (10.4)	117 (9.5)	1,163 (17.5)
Different cognitive test	445 (6.7)	196 (4.2)	63 (2.5)	37 (3.0)	633 (9.5)
Clinical tests
Any clinical tests	6,121 (91.9)	3,584 (76.9)	1,823 (72.6)	811 (65.6)	6396 (96)
Clinical examination/discussed symptoms	5,407 (81.2)	2,147 (46.0)	1,010 (40.2)	460 (37.2)	5,804 (87.1)
Current medications taken	5,115 (76.8)	2,021 (43.4)	969 (38.6)	430 (34.8)	5,605 (84.1)
Family history	4,933 (74.0)	934 (20.0)	283 (11.3)	99 (8.0)	5,457 (81.9)
MRI	2,377 (35.7)	1,162 (24.9)	485 (19.3)	175 (14.2)	3,845 (57.7)
CT scan	1,479 (22.2)	739 (15.8)	336 (13.4)	119 (9.6)	2,460 (36.9)
CSF biomarker testing	373 (5.6)	336 (7.2)	193 (7.7)	77 (6.2)	866 (13.0)
PET amyloid confirmation	336 (5.0)	350 (7.5)	277 (11.0)	137 (11.1)	974 (14.6)
Blood tests
Any blood tests	2,497 (37.5)	1,008 (21.6)	449 (17.9)	207 (16.7)	3,341 (50.2)
*APOE* ɛ4 or any other relevant genetic mutations	592 (8.9)	512 (11.0)	299 (11.9)	134 (10.8)	1,316 (19.8)
Blood tests to rule out other causes*	2,104 (31.6)	621 (13.3)	198 (7.9)	103 (8.3)	2,697 (40.5)
Other blood tests	149 (2.2)	66 (1.4)	20 (0.8)	5 (0.4)	201 (3.0)
Other	158 (2.4)	101 (2.2)	67 (2.7)	40 (3.2)	282 (4.2)
None of these	93 (1.4)	215 (4.6)	229 (9.1)	202 (16.3)	486 (7.3)

ADAS-Cog, Alzheimer’s Disease Assessment Scale– Cognitive Subscale; ANAM, Automated Neuropsychological Assessment Metrics; *APOE* ɛ4, ɛ4 allele of Apolipoprotein E gene; BEHAVE-AD, Behavioral Pathology in Alzheimer’s Disease Rating Scale; CANTAB, Cambridge Neuropsychological Test Automated Battery; CSF, cerebrospinal fluid; CT, computed tomography; GPCOG, General Practitioner Assessment of Cognition; MMSE, Mini-Mental State Examination; MRI, magnetic resonance imaging; PET, positron emission tomography. * These include tests to rule out treatable causes of cognitive decline, such as vitamin B12 deficiency.

### Patient demographics

The majority of the patients were 66 years and older (66– 75 years, 36.3% and 76 + years, 42.9%), and there were no notable differences in the age group distribution across countries or by HCP specialty (data not shown).

### Patient symptoms

#### Symptoms presented at physician appointment

Overall, the most common presenting symptoms (multiple responses were possible) were memory/amnestic (90.0%); followed by physical or behavioral (87.0%), those related to cognitive skills, such as difficulty with problem solving (73.0%), language (71.8%), and disorientation (46.0%), with no notable differences across countries. There were no notable differences by HCP specialty (Supplementary Table 3).

#### Initiation of diagnostic work-up

Overall, the proportion of patients having their first consultation for cognitive symptoms (i.e., incident cases; 42.1%) was similar to the proportion who had had a prior consultation (43.6%; Supplementary Table 4). This was true in all countries except Spain (first consultation, 36.3%; prior consultations, 52.3%). Overall, the proportion of first consultations was higher for PCPs than specialists (50.2% versus 38.1%); this was the case for all countries except China (45.8% versus 50.5%) (Supplementary Table 4).

Investigations for symptoms of cognitive impairment/cognitive complaints were most commonly initiated by the patient or family (57.9%), followed by the participating HCP (29.6%) and a referring physician (11.8%) (Supplementary Table 4). This pattern was different only in the UK, where investigations were most commonly initiated by the patient or family, followed by a referring physician and then the participating HCP. The general trend was the same for PCPs and for specialists; except for specialists in the UK, who reported a referring physician as the second-most common initiator after patient or family (Supplementary Table 4).

Overall, patients made a mean 2.81 visits (median: 2 visits) to investigate cognitive symptoms (Table 1). The number of visits was relatively high in Spain (mean, 3.72; median, 3 visits). A small fraction of the population (3.8% [258/6744]) had > 7 visits; 59% (152/258) of whom were patients under long-term management by the HCP for “general health or another comorbidity”, who had presented with a cognitive complaint. Removal of these outliers had minimal effect on the mean number of visits (which reduced to 2.33), and no effect on the median number of visits (which remained as 2); data not shown.

### Assessments used to investigate symptoms of cognitive impairment/cognitive complaints

Overall, cognitive (e.g., Mini-Mental State Examination [MMSE] [29]) and clinical (e.g., clinical examination and discussions) tests were used in nearly all patients (>95%), with blood tests (i.e., genetic tests for *APOE* ɛ4 or other relevant mutations, or blood tests to rule out treatable causes of cognitive decline such as vitamin B12 deficiency) being used in half (50.2%) (Table 2). The most common cognitive test was MMSE (79.0%); followed by standard psychological/psychiatric evaluations (68.9%), Mini-Cog [30] (39.1%) and Alzheimer’s Disease Assessment Scale– Cognitive Subscale (ADAS-Cog) [31] (23.8%). Other cognitive tests were used in < 20% of people presenting with symptoms/patients. The use of cognitive tests declined by appointments from 88.8% at appointment 1 to 48.9% after three appointments. This decline was also seen for individual tests, except for the CANTAB mobile device test [32] and the Cognigram [33], Cognivue [34], Cognision [35], or Automated Neuropsychological Assessment Metrics (ANAM) device test [36].

The most common clinical test was clinical examination/discussion of symptoms (87.1%) followed by assessment of current medications (84.1%), family history (81.9%), magnetic resonance imaging (MRI; 57.7%), and computed tomography (CT) scan (36.9%). In addition, cerebrospinal fluid (CSF) biomarker and amyloid-positron emission tomography (PET) tests were done in < 15% of patients each. The use of clinical tests also declined by appointment, from 91.9% at appointment 1 to 65.6% after three appointments. The only individual tests that did not show this pattern were CSF (5.6% at appointment 1 and 6.2% after three appointments) and amyloid PET (5.0% at appointment 1 and 11.1% after three appointments), though the rates of ordering both tests remained low (Table 2).

The most common blood tests were blood tests to rule out other causes of cognitive symptoms (i.e., treatable causes of cognitive decline, such as vitamin B12 deficiency; 40.5%), followed by genetic tests for *APOE* ɛ4 or other relevant mutations (19.8%). Similarly as for cognitive and clinical tests, the use of blood tests overall also declined with appointment, from 37.5% at appointment 1 to 16.7% after three appointments; although genetic tests did not show a decline (Table 2).

The frequency and pattern of cognitive, clinical and blood tests were not notably different in the subgroup of incident cases (data not shown).

Overall, PCPs and specialists used tests at similar rates. PCPs used some cognitive tests less frequently than specialists (MMSE: 72.6% versus 82.6%; standard psychological/psychiatric evaluations: 62.2% versus 72.5%; Mini-Cog: 36.0% versus 41.7%; ADAS-Cog: 16.6% versus 28.6%), and the General Practitioner Assessment of Cognition (GPCOG) [37] more frequently than specialists (25.1% versus 14.5%). PCPs also ordered MRIs and CSF biomarker tests less frequently than specialists (47.0% versus 66.1% and 8.1% versus 16.4%, respectively). Amyloid PET and CSF biomarker testing were often ordered at low rates even by specialists (across countries, 5.7% to 28.7% for amyloid PET and 5.0% to 27.3% for CSF testing; Table 3).

**Table 3:** Summary of assessments used to investigate

		All	US		China		UK		France		Germany		Spain	
	Total	PCP	Specialist	PCP	Specialist	PCP	Specialist	PCP	Specialist	PCP	Specialist	PCP	Specialist	PCP	Specialist
		n (%)	n (%)	n (%)	n (%)	n (%)	n (%)	n (%)	n (%)	n (%)	n (%)	n (%)	n (%)	n (%)	n (%)
All	6,525^‡^ (100)	2,482 (100)	4,043 (100)	758 (100)	944 (100)	173 (100)	1,012 (100)	371 (100)	548 (100)	345 (100)	513 (100)	395 (100)	454 (100)	440 (100)	572 (100)
Cognitive tests
Any cognitive tests	6,229 (95.5)	2,298 (92.6)	3,931 (97.2)	727 (95.9)	924 (97.9)	123 (71.1)	953 (94.2)	358 (96.5)	535 (97.6)	319 (92.5)	508 (99.0)	350 (88.6)	450 (99.1)	421 (95.7)	561 (98.1)
Standard psychological/ psychiatric evaluation	4,477 (68.6)	1,544 (62.2)	2,933 (72.5)	452 (59.6)	720 (76.3)	51 (29.5)	542 (53.6)	193 (52.0)	318 (58.0)	238 (60.0)	429 (83.6)	262 (66.3)	407 (89.6)	348 (79.1)	517 (90.4)
MMSE	5,141 (78.8)	1,802 (72.6)	3,339 (82.6)	571 (75.3)	742 (78.6)	72 (41.6)	829 (81.9)	242 (65.2)	396 (72.3)	293 (84.9)	473 (92.2)	242 (61.3)	384 (84.6)	382 (86.8)	515 (90.0)
Mini-Cog	2,577 (39.5)	893 (36.0)	1,684 (41.7)	385 (50.8)	405 (42.9)	53 (30.6)	455 (45.0)	83 (22.4)	245 (44.7)	66 (19.1)	132 (25.7)	112 (28.4)	154 (33.9)	194 (44.1)	293 (51.2)
ADAS-Cog	1,570 (24.1)	412 (16.6)	1,158 (28.6)	103 (13.6)	176 (18.6)	26 (15.0)	369 (36.5)	23 (6.2)	109 (19.9)	26 (7.5)	101 (19.7)	38 (9.6)	94 (20.7)	196 (44.5)	309 (54.0)
GPCOG	1,211 (18.6)	623 (25.1)	588 (14.5)	129 (17.0)	125 (13.2)	73 (42.2)	42 (4.2)	165 (44.5)	74 (13.5)	31 (9.0)	67 (13.1)	50 (12.7)	78 (17.2)	175 (39.8)	202 (35.3)
CANTAB mobile device test	672 (10.3)	248 (10.0)	424 (10.5)	56 (7.4)	87 (9.2)	12 (6.9)	50 (4.9)	14 (3.8)	23 (4.2)	26 (7.5)	53 (10.3)	35 (8.9)	54 (11.9)	105 (23.9)	157 (27.4)
Cognigram, Cognivue, Cognision or ANAM device tests	784 (12.0)	261 (10.5)	523 (12.9)	65 (8.6)	105 (11.1)	12 (6.9)	57 (5.6)	14 (3.8)	53 (9.7)	29 (8.4)	69 (13.5)	36 (9.1)	67 (14.8)	105 (23.9)	172 (30.1)
BEHAVE-AD	1,161 (17.8)	375 (15.1)	786 (19.4)	91 (12.0)	176 (18.6)	32 (18.5)	170 (16.8)	24 (6.5)	80 (14.6)	28 (8.1)	69 (13.5)	34 (8.6)	63 (13.9)	166 (37.7)	228 (39.9)
Different cognitive test	626 (9.6)	179 (7.2)	447 (11.1)	34 (4.5)	102 (10.8)	4 (2.3)	34 (3.4)	34 (9.2)	96 (17.5)	23 (6.7)	84 (16.4)	56 (14.2)	93 (20.5)	28 (6.4)	38 (6.6)
Clinical tests
Any clinical tests	6,261 (96.0)	2,342 (94.4)	3,919 (96.9)	704 (92.9)	894 (94.7)	172 (99.4)	997 (98.5)	355 (95.7)	539 (98.4)	328 (95.1)	501 (97.7)	367 (92.9)	443 (97.6)	416 (94.5)	545 (95.3)
Clinical examination/ discussed symptoms	5,672 (86.9)	2,129 (85.8)	3,543 (87.6)	652 (86.0)	793 (84.0)	122 (70.5)	876 (86.6)	336 (90.6)	495 (90.3)	302 (87.5)	470 (91.6)	333 (84.3)	418 (92.1)	384 (87.3)	491 (85.8)
Current medications taken	5,483 (84.0)	2,050 (82.6)	3,433 (84.9)	631 (83.2)	789 (83.6)	117 (67.6)	851 (84.1)	342 (92.2)	457 (83.4)	280 (81.2)	443 (86.4)	298 (75.4)	394 (86.8)	382 (86.8)	499 (87.2)
Family history	5,337 (81.8)	2,019 (81.3)	3,318 (82.1)	599 (79.0)	766 (81.1)	157 (90.8)	811 (80.1)	288 (77.6)	414 (75.5)	287 (83.2)	448 (87.3)	310 (78.5)	390 (85.9)	378 (85.9)	489 (85.5)
MRI	3,837 (58.8)	1,166 (47.0)	2,671 (66.1)	379 (50.0)	551 (58.4)	29 (16.8)	775 (76.6)	75 (20.2)	275 (50.2)	250 (72.5)	392 (76.4)	211 (53.4)	338 (74.4)	222 (50.5)	340 (59.4)
CT scan	2,446 (37.5)	899 (36.2)	1,547 (38.3)	249 (32.8)	321 (34.0)	45 (26.0)	390 (38.5)	122 (32.9)	235 (42.9)	107 (31.0)	136 (26.5)	105 (26.6)	136 (30.0)	271 (61.6)	329 (57.5)
CSF biomarker testing	866 (13.3)	202 (8.1)	664 (16.4)	49 (6.5)	134 (14.2)	1 (0.6)	51 (5.0)	10 (2.7)	77 (14.1)	14 (4.1)	122 (23.8)	35 (8.9)	124 (27.3)	93 (21.1)	156 (27.3)
PET amyloid confirmation	970 (14.9)	306 (12.3)	664 (16.4)	81 (10.7)	174 (18.4)	14 (8.1)	58 (5.7)	19 (5.1)	79 (14.4)	50 (14.5)	97 (18.9)	44 (11.1)	92 (20.3)	98 (22.3)	164 (28.7)
Blood tests
Any blood tests	3,329 (51.0)	1,301 (52.4)	2,028 (50.2)	373 (49.2)	512 (54.3)	43 (24.9)	334 (33.0)	232 (62.5)	253 (46.2)	163 (47.2)	266 (51.9)	209 (52.9)	255 (56.2)	281 (63.9)	408 (71.3)
*APOE* ɛ4 or any other relevant genetic mutations	1,312 (20.1)	431 (17.4)	881 (21.8)	145 (19.1)	231 (24.5)	22 (12.7)	137 (13.5)	28 (7.5)	82 (15.0)	56 (16.2)	88 (17.2)	52 (13.2)	113 (24.9)	128 (29.1)	230 (40.2)
Blood tests to rule out other causes*	2,687 (41.2)	1,092 (44.0)	1,595 (39.5)	288 (38.0)	399 (42.3)	32 (18.5)	239 (23.6)	218 (58.8)	216 (39.4)	134 (38.8)	233 (45.4)	183 (46.3)	193 (42.5)	237 (53.9)	315 (55.1)
Other blood tests	199 (3.0)	58 (2.3)	141 (3.5)	7 (0.9)	59 (6.3)	N/A	16 (1.6)	12 (3.2)	19 (3.5)	N/A	26 (5.1)	8 (2.0)	18 (4.0)	31 (7.0)	3 (0.5)
Other	282 (4.3)	134 (5.4)	148 (3.7)	41 (5.4)	51 (5.4)	N/A	1 (0.1)	35 (9.4)	24 (4.4%)	17 (4.9)	35 (6.8)	13 (3.3)	25 (5.5)	28 (6.4)	12 (2.1)
None of these	486 (7.4)	244 (9.8)	242 (6.0)	64 (8.4)	80 (8.5)	1 (0.6)	13 (1.3)	58 (15.6)	36 (6.6)	39 (11.3)	29 (5.7)	36 (9.1)	27 (5.9)	46 (10.5)	57 (10.0)

ADAS-Cog, Alzheimer’s Disease Assessment Scale– Cognitive Subscale; ANAM, Automated Neuropsychological Assessment Metrics; *APOE* ɛ4, ɛ4 allele of Apolipoprotein E gene; BEHAVE-AD, Behavioral Pathology in Alzheimer’s Disease Rating Scale; CANTAB, Cambridge Neuropsychological Test Automated Battery; CSF, cerebrospinal fluid; CT, computed tomography; GPCOG, General Practitioner Assessment of Cognition; MMSE, Mini-Mental State Examination; MRI, magnetic resonance imaging; N/A, not applicable (implies that the particular test was not applicable for PCP/specialist in that country); PET, positron emission tomography. ^‡^ Surveys filled out by nurses are excluded from tables stratified by HCP, hence the total N is not consistent across all tables. * These include tests to rule out treatable causes of cognitive decline.

The pattern of cognitive tests was similar in individual countries, with a few notable exceptions (Table 3): a relatively low proportion of PCPs (71.1%) used a cognitive test in China; the GPCOG was used at a similar rate as MMSE by PCPs in China (42.2% versus 41.6%), and psychological/psychiatric evaluations were used at a similar frequency as MMSE by specialists in Germany (89.6% versus 84.6%) and Spain (90.4% versus 90.0%). PCPs in China had low testing rates for the following clinical tests: clinical examinations/discussed symptoms (70.5%), current medication assessments (67.6%), MRIs (16.8%), and CSF testing (0.6%); specialists in China ordered PET amyloid tests at a low frequency (5.7%); PCPs in the UK ordered MRIs, CSF testing, and PET amyloid tests at low frequencies (20.2%, 2.7%, and 5.1%, respectively); and specialists in the UK ordered MRIs at a low frequency (50.2%).

There were also some variations from the overall pattern for blood tests (Table 3): in China a low frequency of blood tests overall was ordered by both PCPs (24.9%) and specialists (33.0%); and a high frequency was ordered by PCPs (62.5%) in the UK, and PCPs and specialists in Spain (63.9% and 71.3%; note that Spain had the highest frequency of academic/tertiary/regional hospitals, hence high testing frequency may be expected). *APOE* ɛ4 and other genetic blood tests were ordered at a low frequency by PCPs in the UK (7.5%), and at a high frequency by specialists in Spain (40.2%). Blood tests to rule out treatable causes of cognitive decline were ordered at low frequencies by both PCPs (18.5%) and specialists (23.6%) in China, and at a high frequency by PCPs in the UK (58.8%).

### Outcomes

#### Summary of outcomes

Overall, less than half (47.0%) of the patients received a diagnosis for their cognitive impairments or complaints (ranging from 36.1% in France to 65.6% in China); 23.4% were referred to another specialty (from 14.0% in China to 33.5% in France); 21.2% were advised to watch and wait (from 13.5% in China to 24.8% in the UK); and 6.3% were told there was nothing significantly wrong/there was no diagnosis to give (from 1.4% in China to 8.5% in the US; Table 4). Of the diagnoses given, the majority (52.1%) were AD (ranging from 33.8% in the UK to 65.6% in China); 17.4% were vascular dementia (from 11.3% in the US to 23.8% in Spain); and 15.1% were non-etiologic diagnoses such as MCI or subjective cognitive decline (from 7.0% in France to 27.7% in the UK). Small proportions were dementia linked to Parkinson’s disease and Lewy body dementia.

**Table 4:** Summary of appointment results and diagnoses, by country

	All n (%)	US n (%)	China n (%)	UK n (%)	France n (%)	Germany n (%)	Spain n (%)
All	6,744 (100.0)	1,738 (100.0)	1,204 (100.0)	1,056 (100.0)	871 (100.0)	852 (100.0)	1,023 (100.0)
Result of appointment with the doctor
Patient referred to another specialty for further tests and/or diagnosis	1,577 (23.4)	386 (22.2)	168 (14.0)	311 (29.5)	292 (33.5)	163 (19.1)	257 (25.1)
Patient given a diagnosis	3,172 (47.0)	785 (45.2)	790 (65.6)	408 (38.6)	314 (36.1)	426 (50.0)	449 (43.9)
Patient told there was nothing significantly wrong/no diagnosis to give	422 (6.3)	147 (8.5)	17 (1.4)	63 (6.0)	66 (7.6)	61 (7.2)	68 (6.6)
Watch and wait	1,429 (21.2)	392 (22.6)	162 (13.5)	262 (24.8)	181 (20.8)	193 (22.7)	239 (23.4)
Other	144 (2.1)	28 (1.6)	67 (5.6)	12 (1.1)	18 (2.1)	9 (1.1)	10 (1.0)
Diagnosis given
All	3,172 (100.0)	785 (100.0)	790 (100.0)	408 (100.0)	314 (100.0)	426 (100.0)	449 (100.0)
Alzheimer’s disease	1,654 (52.1)	431 (54.9)	518 (65.6)	138 (33.8)	184 (58.6)	196 (46.0)	187 (41.6)
Vascular dementia	552 (17.4)	89 (11.3)	121 (15.3)	90 (22.1)	47 (15.0)	98 (23.0)	107 (23.8)
Lewy Body dementia	140 (4.4)	41 (5.2)	8 (1.0)	20 (4.9)	20 (6.4)	16 (3.8)	35 (7.8)
Dementia linked to Parkinson’s disease	245 (7.7)	49 (6.2)	65 (8.2)	31 (7.6)	22 (7.0)	44 (10.3)	34 (7.6)
Descriptive diagnosis of symptoms (i.e., MCI or SCD)*	478 (15.1)	147 (18.7)	68 (8.6)	113 (27.7)	22 (7.0)	55 (12.9)	73 (16.3)
Other	103 (3.2)	28 (3.6)	10 (1.3)	16 (3.9)	19 (6.1)	17 (4.0)	13 (2.9)

MCI, mild cognitive impairment; SCD, subjective cognitive decline; UK, United Kingdom; US, United States of America *Refers to non-etiologic diagnoses of symptoms.

Stratified by specialty, PCPs made referrals more frequently than specialists (40.7% versus 13.1%), and conversely, specialists gave a diagnosis more frequently than PCPs (57.2% versus 29.5%). Similar proportions of PCPs and specialists advised patients to watch and wait (20.7% versus 21.5%), or told patients there was nothing significantly wrong/no diagnosis to give (6.6% versus 6.2%). Similar patterns were seen in all countries, with some variability in rates; the difference between specialties was most marked in China, where 75.5% of specialists gave a diagnosis versus 13.5% of PCPs (Supplementary Table 5). Overall, AD was diagnosed at a similar rate by PCPs and specialists (48.1% versus 55.6%); the same was true for other diagnoses. Exceptions were seen only in China, where PCPs diagnosed dementia related to Parkinson’s disease more frequently than specialists (30.8% versus 7.5%), and vascular dementia less frequently than specialists (3.8% versus 15.7%) (Supplementary Table 5). There were no notable differences in the subgroup of incident cases (data not shown).

As shown in the Sankey diagram in Supplementary Figure 1, most referrals to participating HCPs came from PCPs (58.8%). With regards to the pattern of referrals ‘outwards’ (Supplementary Figure 2), participating HCPs made referrals primarily to memory specialists (35.4%) and neurologists (30.7%), with smaller proportions to other specialties (geriatricians/psychiatrists/’other’/PCPs and radiologists).

#### Time to final diagnosis

As shown in the Kaplan– Meier curve of overall time to final diagnosis (Supplementary Figure 3), the probability of *not* receiving a diagnosis declined steeply (from 1 to < 0.1) in the first 5 months, and thereafter declined gradually till Month 20. In the analysis of factors influencing the risk of not receiving a final diagnosis (Table 5), the risk was found to be significantly higher for PCPs versus specialists (HR, 1.1). In comparison to the US, the risk was higher in China (HR, 1.412) and Germany (HR, 1.163) and lower in the UK (HR, 0.685), France (HR, 0.73) and Spain (HR, 0.784). Presenting symptoms, age group and interaction type had no influence.

**Table 5:** Analysis of risk of not receiving a final diagnosis, all countries

Parameter	Comparison	Analysis of maximum likelihood estimates	
		Pr> χ^2^	Hazard ratio (95% CI)
Physician type	PCP versus specialist	0.0427	**1.1 (1.003, 1.207)**
Age group	45– 55 y versus 45 and younger	0.9963	0.999 (0.683, 1.461)
	56– 65 y versus 45 and younger	0.8967	1.024 (0.72, 1.455)
	66– 75 y versus 45 and younger	0.8223	1.04 (0.738, 1.467)
	76 + y versus 45 and younger	0.755	1.056 (0.749, 1.488)
	Unknown versus 45 and younger	0.4547	1.486 (0.526, 4.199)
Country	China versus US	<0.0001	**1.412 (1.265, 1.575)**
	UK versus US	<0.0001	**0.685 (0.596, 0.788)**
	France versus US	<0.0001	**0.73 (0.638, 0.836)**
	Germany versus US	0.0144	**1.163 (1.031, 1.313)**
	Spain versus US	<0.0001	**0.784 (0.694, 0.885)**
Interaction type	Survey physician versus patient/family	0.2575	0.949 (0.866, 1.039)
	Referral versus patient/family	0.8401	1.012 (0.9, 1.138)
	Other/Missing vs patient/family	0.2724	0.743 (0.437, 1.263)
Presenting symptoms	Physical/behavioral	0.4783	0.958 (0.85, 1.079)
	Cognitive skills	0.6418	0.979 (0.897, 1.07)
	Language	0.8593	0.992 (0.911, 1.081)
	Disorientation	0.6691	1.017 (0.943, 1.097)
	Memory/amnestic	0.0801	0.887 (0.775, 1.015)

CI, confidence interval; PCP, primary care physician; UK, United Kingdom; US, United States of America. *Notes:* a) Surveys filled out by nurses are excluded for all the regressions and KM curves containing specialty break-down. b) Patients without a valid value of the time variable are excluded. c) Patients’ time are rounded to the next integer month (<1 month will have the event at 1 month, 1.5 month will have the event at 2 months). d) Significant results are shaded in green.

## DISCUSSION

### Summary of findings

This real-world, cross-sectional study analyzed extracted data for 6,744 patients across six countries. There were several commonalities across countries: patients had a similar distribution across age groups; the most common symptoms at presentation were cognitive (memory/amnestic), followed by physical or behavioral; and investigations were most commonly initiated by the patient or family. Clinical and cognitive tests were used in nearly all patients (with the exception that PCPs in China used cognitive tests at a lower rate [∼70%]), and blood tests in half (except in China, where they were used in about a third of patients). Across countries, the most common cognitive test was the MMSE (except in China, where PCPs used the GPCOG at a similar rate); the most common clinical tests were clinical examination/discussion of symptoms and assessment of current medications; and the most common blood tests were those used to rule out treatable causes of cognitive decline.

Though the survey did not explore the use of guidelines, the pattern of testing over time appears to indicate broad compliance. For example, clinical and cognitive tests were used at higher frequency at earlier visits, and PET/CSF biomarker testing at higher frequency at later visits. This is in line with recent guidelines ([39], and summarized in [40]), which advise a tiered approach: cognitive deficits should be confirmed, and physiological causes for cognitive decline ruled out, before biomarker tests are conducted. Further, though the use of amyloid PET and CSF biomarker testing increased as the diagnostic pathway progressed, rates remained low even after the 3^rd^ appointment (11.1% overall for amyloid PET and 6.2% for CSF testing). By country, rates were lowest in China, where amyloid PET and CSF testing was ordered by 5.7% and 5.0% of specialists, respectively. Though our survey did not explore which factors may have influenced these low rates, reimbursement is likely to have been one. Amyloid PET is reimbursed in Spain, but not in France, Germany, the UK, or the US [41–44], and not fully covered in China [45]; CSF testing is reimbursed in France, Germany, and the UK, but not in Spain or the US [42, 46]. In addition to reimbursement issues, cost, capacity limitations and inconclusiveness of test results have been reported as barriers to ordering amyloid PET [2, 25, 47–49], and invasiveness of lumbar puncture as the main barrier to ordering CSF biomarker tests [2, 25].

In keeping with data on high rates of undiagnosed dementia globally [14], we found that diagnosis rates were low. Only about half of the patients received diagnoses, and about half of these were AD. Substantial proportions of patients were told to ‘watch and wait’ (21.2%), or referred to another specialty (23.4%). The probability of not receiving a diagnosis declined steeply in the first 5 months from the first presentation/test, and thereafter very gradually till month 20. The only factors that influenced the risk of not receiving a diagnosis were HCP type (risk being significantly higher for PCPs) and region (risk being significantly higher in China and Germany than in the US; and significantly lower in the UK, France, and Spain than in the US).

In a similar survey done across multiple countries (including US, UK, France, Germany, and Spain; *n* = 1365 [25]), 71% of the HCPs reported performing initial cognitive testing for patients with cognitive complaints/impairments, which is somewhat lower than in this study (88% at the first appointment). Judge et al. [25], as well as a different multi-country physician survey (including US, UK, France, and Germany; *n* = 1,086 [26]), found the MMSE to be the most commonly used cognitive test, as in this study. Despite this, an analysis of 111,125 dementia patients in a US database [50] found that only 11% of electronic health records had documented cognitive measures (in contrast, nearly all [1,651 of 1,702, or 97%] of the HCPs in our US sample reported using a cognitive test to investigate patients [Supplementary Table 6]). Podhorna et al. [26] also reported concerns raised by family members or caregivers as the most common reason for consultation. Similar to this study, another multi-country survey of patients with dementia (including UK, France, Spain, and China; *n* = 548 [27]) found AD to be the most common diagnosis.

In a smaller physician survey in the US ([51]; *n* = 150) some practice patterns were similar to those in this study (e.g., PCPs and specialists ordered rule-out blood tests at similar rates), while others were not (e.g., PCPs ordered CT scans more frequently and MRI less frequently than specialists). In contrast to findings in this study that presenting symptoms do not influence time to diagnosis, an Australian study [52] in young-onset dementia (i.e., with symptom onset before 65 years; *n* = 88) found time to diagnosis was significantly longer in participants presenting with MCI or depression. Overall, these observations support earlier reports [21–23] on heterogeneity in the diagnostic pathway.

### Limitations

The authors acknowledge certain limitations of this study. There was no attempt to verify the data extracted from patient charts. We cannot be sure how representative the physician sample in each country was, since the sample is likely to be prone to selection bias. Therefore, these observations may not be generalizable to all HCPs and countries. Similarly, our survey did not explore aspects such as the impact of clinical guidelines, if followed; the organization of memory clinics within each country; or the influence of funding for services or investigations (which differs across countries) on the use of tests such as CSF and amyloid PET. Also, other factors which were not explored in our study may influence the time taken to receive a diagnosis, such as level of concern raised by patients, caregiver, or family members [53] and differences in healthcare systems between participating countries [26]; future studies should investigate the influence of these parameters.

Others have emphasized the need for timely detection and diagnosis of patients with cognitive impairment/complaints, e.g., through screening [54, 55]. Challenges include distinguishing pathological cognitive decline from normal aging, especially for PCPs [51], patient reluctance to disclose symptoms, the lack of universally accepted confirmatory biomarker tests and a standard diagnostic pathway, and the current paucity of treatment options [28]. As individual tests such as MRI have their limitations [56], a standard diagnostic pathway should include a variety of clinical, cognitive, and biomarker tests. An earlier analysis found the US healthcare system to have capacity constraints, mainly in terms of insufficient specialists, but also in terms of access to imaging and to infusion centers [57]. With recent regulatory approvals [16], DMTs for AD are expected to become widely available in the near future, which will likely lead to more demand for confirmatory diagnostic tests such as PET and CSF. To identify resource constraints and data gaps and enable patients to access care in a streamlined way in the future, it is important to understand current diagnostic pathways in greater detail.

### Conclusion

This cross-sectional study of patient data extracted by HCPs offers a snapshot of the diagnostic pathway for patients with cognitive impairments or complaints across six countries, and highlights commonalities as well as differences in clinical practice. While most recent studies have focused on high-income countries in Europe and North America [25, 26, 28], our study also provides insights into the diagnostic pathway in China. Our data indicate that, across countries, a high proportion of patients (about 45%) are referred to another specialty or told to ‘watch and wait’. Further, substantial improvements can be made across countries in the use of amyloid PET and CSF testing. Finally, to allow AD patients to be identified and treated as early as possible, the research community should focus its efforts on further defining biomarkers for those at risk for AD. Though a biomarker test may not be needed when the clinical diagnosis is clear, where it is uncertain, an easily accessible biomarker test may avoid many patients being given “watch and wait” advice. Equally important, regulatory agencies should work towards dismantling barriers such low testing capacity and reimbursement challenges. Encouragingly, there have been global efforts to address the last point: the French National Authority has offered a positive opinion on reimbursement of amyloid tracer [41], the US Centers for Medicare & Medicaid Services have opened a consultation to expand its coverage of amyloid PET testing [58], and the AMYPAD study has been initiated in Europe to investigate the clinical utility and cost-effectiveness of amyloid PET [59].

## Supplementary Material

Supplementary Material

## Data Availability

The data supporting the findings of this study are available on request from the corresponding author.
