# Supplementary material for: Current Diagnostic Pathways for Alzheimer’s Disease: A Cross-Sectional Real-World Study Across Six Countries
Source: J Alzheimers Dis Rep. 2023 Jun 29;7(1):659–74. doi: 10.3233/ADR230007 (PMC10357118; doi:10.3233/ADR230007)
Supplement: Supplementary Material [file adr-7-adr230007-s001.pdf]

Supplementary Material

Current Diagnostic Pathways for Alzheimer's Disease: A Cross-Sectional Real-World Study Across Six Countries

Supplementary Table 1. Data management standard operating procedures

Stage	Short description (Responsibility)
1. Survey link	Once the survey link was scripted, it was quality checked against the questionnaire by two methods:<br>1. Manually, by going through the link (Scripting Team)<br>2. By preparing a data validation script to check the data thoroughly for each respondent and for each question in the survey. Dummy data were generated, and the data validation script was checked to verify all logic was followed as per questionnaire before the survey was launched to collect the live sample (Data Processing Team)
2. Live data	Live data collected during fieldwork were also quality checked using the same data validation script as above at the following stages (Data Processing Team):<br>1. Soft launch data; after achieving 10% of total sample<br>2. Interim data; after achieving 50% of total sample<br>3. Final data; after achieving the total sample

Supplementary Table 2. Summary of healthcare providers by specialty and practice setting, by country

	All n (%)	US n (%)	China n (%)	UK n (%)	France n (%)	Germany n (%)	Spain n (%)
**All**	1,694 (100.0)	455 (100.0)	300 (100.0)	255 (100.0)	233 (100.0)	210 (100.%)	241 (100.0)
**Physician specialty***							
Primary care physician/general practice physician	657 (38.8)	202 (44.4)	50 (16.7)	101 (39.6)	101 (43.3)	97 (46.2)	106 (44.0)
Geriatrician	207 (12.2)	50 (11.0)	50 (16.7)	30 (11.8)	34 (14.6)	13 (6.2)	30 (12.4)
Neurologist	403 (23.8)	100 (22.0)	100 (33.3)	50 (19.6)	48 (20.6)	50 (23.8)	55 (22.8)
Psychiatrist	402 (23.7)	103 (22.6)	100 (33.3)	49 (19.2)	50 (21.5)	50 (23.8)	50 (20.7)
Nurse from a memory clinic	25 (1.5)	N/A	N/A	25 (9.8)	N/A	N/A	N/A
**Practice type**							
Academic/tertiary/regional hospital	583 (34.4)	95 (20.9)	166 (55.3)	69 (27.1)	56 (24.0)	53 (25.2)	144 (59.8)
Large general/regional hospitals	228 (13.5)	57 (12.5)	15 (5.0)	69 (27.1)	21 (9.0)	37 (17.6)	29 (12.0)
Community/ secondary hospital	274 (16.2)	75 (16.5)	89 (29.7)	36 (14.1)	44 (18.9)	7 (3.3)	23 (9.5)
Small community/ primary hospital (<100 beds)	67 (4.0)	6 (1.3)	30 (10.0)	8 (3.1)	9 (3.9)	2 (1.0)	12 (5.0)
Office practice	542 (32.0)	222 (48.8)	N/A	73 (28.6)	103 (44.2)	111 (52.9)	33 (13.7)

N/A, not applicable; UK, United Kingdom; US, United States of America

* PCPs and nurses were required to spend at least 75% of their time in clinical practice and seeing at least three relevant patients per month, whereas specialists had to spend at least 60% of their time in practice seeing at least five patients per month.

Supplementary Table 3. Summary of symptoms presented at physician appointment, by country and physician specialty

	All*	All		US		China		UK		France		Germany		Spain	
	n (%)	PCP n (%)	Specialist n (%)	PCP n (%)	Specialist n (%)	PCP n (%)	Specialist n (%)	PCP n (%)	Specialist* n (%)	PCP n (%)	Specialist n (%)	PCP n (%)	Specialist n (%)	PCP n (%)	Specialist n (%)
Any	6,607 (100.0)	2,550 (100.0)	4,057 (100.0)	784 (100.0)	954 (100.0)	192 (100.0)	1,012 (100.0)	371 (100.0)	548 (100.0)	358 (100.0)	513 (100.0)	398 (100.0)	454 (100.0)	447 (100.0)	576 (100.0)
Physical/ behavioral	5,748 (87.0)	2,168 (85.0)	3,580 (88.2)	645 (82.3)	818 (85.7)	183 (95.3)	956 (94.5)	311 (83.8)	471 (85.9)	314 (87.7)	435 (84.8)	345 (86.7)	387 (85.2)	370 (82.8)	513 (89.1)
Cognitive skills**	4,823 (73.0)	1,807 (70.9)	3,016 (74.3)	568 (72.4)	754 (79.0)	127 (66.1)	757 (74.8)	264 (71.2)	348 (63.5)	255 (71.2)	372 (72.5)	302 (75.9)	358 (78.9)	291 (65.1)	427 (74.1)
Language	4,746 (71.8)	1,806 (70.8)	2,940 (72.5)	545 (69.5)	715 (74.9)	138 (71.9)	677 (66.9)	249 (67.1)	373 (68.1)	268 (74.9)	382 (74.5)	291 (73.1)	355 (78.2)	315 (70.5)	438 (76.0)
Disorientation	3,036 (46.0)	1,100 (43.1)	1,936 (47.7)	299 (38.1)	439 (46.0)	99 (51.6)	541 (53.5)	147 (39.6)	223 (40.7)	192 (53.6)	255 (49.7)	151 (37.9)	194 (42.7)	212 (47.4)	284 (49.3)
Memory/ amnestic	5,943 (90.0)	2,257 (88.5)	3,686 (90.9)	701 (89.4)	845 (88.6)	168 (87.5)	938 (92.7)	325 (87.6)	514 (93.8)	326 (91.1)	457 (89.1)	352 (88.4)	414 (91.2)	385 (86.1)	518 (89.9)
Other symptoms	97 (1.5)	25 (1.0)	72 (1.8)	5 (0.6)	23 (2.4)	N/A	4 (0.4)	5 (1.3)	16 (2.9)	7 (2.0)	9 (1.8)	3 (0.8)	12 (2.6)	5 (1.1)	8 (1.4)

N/A, not applicable (implies that the particular test was not applicable for PCP/specialist in that country); PCP, primary care physician; UK, United Kingdom; US, United States of America.
*Patients of nurses were not included; therefore, N for totals is not the same as in Table 2
**These include difficulties with logic, problem solving, decision-making, concentration, planning, attention, executive function, organizing and/or sequencing, and issues controlling finances
Note that more than one response option was possible; hence totals for each column will exceed 100%.

Supplementary Table 4. Summary of patients with symptoms of cognitive impairment/cognitive complaints by type of consultation and initiator, by country and specialty

	All			US		China		UK		France		Germany		Spain	
	**All* n (%)**	**PCP n (%)**	**Specialist n (%)***	**PCP n (%)**	**Specialist n (%)***	**PCP n (%)**	**Specialist n (%)***	**PCP n (%)**	**Specialist n (%)***	**PCP n (%)**	**Specialist n (%)***	**PCP n (%)**	**Specialist n (%)***	**PCP n (%)**	**Specialist n (%)***
**All**	6,607 (100.0)	2,550 (100.0)	4,057 (100.0)	784 (100.0)	954 (100.0)	192 (100.0)	1,012 (100.0)	371 (100.0)	548 (100.0)	358 (100.0)	513 (100.0)	398 (100.0)	454 (100.0)	447 (100.0)	576 (100.0)
**First consultation for symptoms of cognitive impairment/cognitive complaints:**															
Yes	2,823 (42.7)	1,279 (50.2)	1,544 (38.1)	404 (51.5)	321 (33.6)	88 (45.8)	511 (50.5)	217 (58.5)	167 (30.5)	188 (52.5)	205 (40.0)	180 (45.2)	171 (37.7)	202 (45.2)	169 (29.3)
No	2,939 (44.5)	1,148 (45.0)	1,791 (44.1)	348 (44.4)	448 (47.0)	86 (44.8)	439 (43.4)	146 (39.4)	193 (35.2)	149 (41.6)	192 (37.4)	198 (49.7)	205 (45.2)	221 (49.4)	314 (54.5)
Missing/ unknown	845 (12.8)	123 (4.8)	722 (17.8)	32 (4.1)	185 (19.4)	18 (9.4)	62 (6.1)	8 (2.2)	188 (34.3)	21 (5.9)	116 (22.6)	20 (5.0)	78 (17.2)	24 (5.4)	93 (16.1)
**Initiation of interaction for symptoms of cognitive impairment/cognitive complaints by:**															
Survey physician	1,996 (30.2)	821 (32.2)	1,175 (29.0)	229 (29.2)	270 (28.3)	91 (47.4)	351 (34.7)	93 (25.1)	134 (24.5)	135 (37.7)	162 (31.6)	144 (36.2)	104 (22.9)	129 (28.9)	154 (26.7)
Referral from another physician	673 (10.2)	54 (2.1)	619 (15.3)	13 (1.7)	165 (17.3)	1 (0.5)	31 (3.1)	4 (1.1)	175 (31.9)	11 (3.1)	106 (20.7)	10 (2.5)	59 (13.0)	15 (3.4)	83 (14.4)
Patient/family	3,892 (58.9)	1,655 (64.9)	2,237 (55.1)	541 (69.0)	515 (54.0)	87 (45.3)	621 (61.4)	273 (73.6)	235 (42.9)	210 (58.7)	243 (47.4)	243 (61.1)	286 (63.0)	301 (67.3)	337 (58.5)
Missing/ unknown	46 (0.7)	20 (0.8)	26 (0.6)	1 (0.1)	4 (0.4)	13 (6.8)	9 (0.9)	1 (0.3)	4 (0.7)	2 (0.6)	2 (0.4)	1 (0.3)	5 (1.1)	2 (0.4)	2 (0.3)

PCP, primary care physician; UK, United Kingdom; US, United States of America.
*Patients of nurses were not included; therefore, N for totals is not the same as N for PCP/specialists

Supplementary Table 5. Summary of appointment results and diagnoses, by country and physician specialty

	All	All		US		China		UK		France		Germany		Spain	
		PCP n (%)	Special-ist n (%)	PCP n (%)	Special-ist n (%)	PCP n (%)	Special-ist n (%)	PCP n (%)	Special-ist n (%)*	PCP n (%)	Special-ist n (%)	PCP n (%)	Special-ist n (%)	PCP n (%)	Special-ist n (%)
**All**	6,607* (100.0)	2,550 (100.0)	4,057 (100.0)	784 (100)	954 (100)	192 (100)	1,012 (100)	371 (100)	548 (100)	358 (100)	513 (100)	398 (100)	454 (100)	447 (100)	576 (100)
**Result of appointment with the doctor**															
Patient referred to another specialty for further tests and/or diagnosis	1,571 (23.8)	1,038 (40.7)	533 (13.1)	258 (32.9)	128 (13.4)	97 (50.5)	71 (7.0)	226 (60.9)	79 (14.4)	172 (48.0)	120 (23.4)	113 (28.4)	50 (11.0)	172 (38.5)	85 (14.8)
Patient given a diagnosis	3,073 (46.5)	753 (29.5)	2,320 (57.2)	277 (35.3)	508 (53.2)	26 (13.5)	764 (75.5)	59 (15.9)	250 (45.6)	86 (24.0)	228 (44.4)	157 (39.4)	269 (59.3)	148 (33.1)	301 (52.3)
Patient told there was nothing significantly wrong/no diagnosis to give	419 (6.3)	169 (6.6)	250 (6.2)	71 (9.1)	76 (8.0)	3 (1.6)	14 (1.4)	19 (5.1)	41 (7.5)	32 (8.9)	34 (6.6)	27 (6.8)	34 (7.5)	17 (3.8)	51 (8.9)
Watch and wait	1,402 (21.2)	528 (20.7)	874 (21.5)	169 (21.6)	223 (23.4)	30 (15.6)	132 (13.0)	63 (17.0)	172 (31.4)	64 (17.9)	117 (22.8)	96 (24.1)	97 (21.4)	106 (23.7)	133 (23.1)
Other	142 (2.1)	62 (2.4)	80 (2.0)	9 (1.1)	19 (2.0)	36 (18.8)	31 (3.1)	4 (1.1)	6 (1.1)	4 (1.1)	14 (2.7)	5 (1.3)	4 (0.9)	4 (0.9)	6 (1.0)
**Diagnosis given**															
**All**	3,073 (100)	753 (100)	2320 (100)	277 (100)	508 (100)	26 (100)	764 (100)	59 (100)	250 (100)	86 (100)	228 (100)	157 (100)	269 (100)	148 (100)	301 (100)
Alzheimer's disease	1,651 (53.7)	362 (48.1)	1289 (55.6)	154 (55.6)	277 (54.5)	15 (57.7)	503 (65.8)	25 (42.4)	110 (44.0)	46 (53.5)	138 (60.5)	69 (43.9)	127 (47.2)	53 (35.8)	134 (44.5)
Vascular dementia	530 (17.2)	142 (18.9)	388 (16.7)	27 (9.7)	62 (12.2)	1 (3.8)	120 (15.7)	15 (25.4)	53 (21.2)	16 (18.6)	31 (13.6)	47 (29.9)	51 (19.0)	36 (24.3)	71 (23.6)
Lewy Body dementia	139 (4.5)	36 (4.8)	103 (4.4)	11 (4.0)	30 (5.9)	N/A	8 (1.0)	2 (3.4)	17 (6.8)	5 (5.8)	15 (6.6)	6 (3.8)	10 (3.7)	12 (8.1)	23 (7.6)
Dementia linked to Parkinson's disease	245 (8.0)	64 (8.5)	181 (7.8)	20 (7.2)	29 (5.7)	8 (30.8)	57 (7.5)	4 (6.8)	27 (10.8)	7 (8.1)	15 (6.6)	12 (7.6)	32 (11.9)	13 (8.8)	21 (7.0)
Descriptive diagnosis of symptoms (i.e., MCI or SCD)	406 (13.2)	130 (17.3)	276 (11.9)	57 (20.6)	90 (17.7)	2 (7.7)	66 (8.6)	11 (18.6)	30 (12.0)	8 (9.3)	14 (6.1)	21 (13.4)	34 (12.6)	31 (20.9)	42 (14.0)
Other	102 (3.3)	19 (2.5)	83 (3.6)	8 (2.9)	20 (3.9)	N/A	10 (1.3)	2 (3.4)	13 (5.2)	4 (4.7)	15 (6.6)	2 (1.3)	15 (5.6)	3 (2.0)	10 (3.3)

MCI, mild cognitive impairment; N/A, not applicable (implies that the response or category was not applicable for PCP/specialist in that country); PCP, primary care physician; SCD, subjective cognitive decline; UK, United Kingdom; US, United States of America.
*Patients of nurses were not included; therefore, N for totals is not the same as N for PCP/specialists.

Supplementary Figure 1. Referral pattern: Flow of patients to the participating healthcare provider by specialty, all countries. A Sankey diagram is shown representing the flow of patients from referring healthcare providers, by specialty (left side), to the healthcare providers participating in the survey, by specialty (right side).

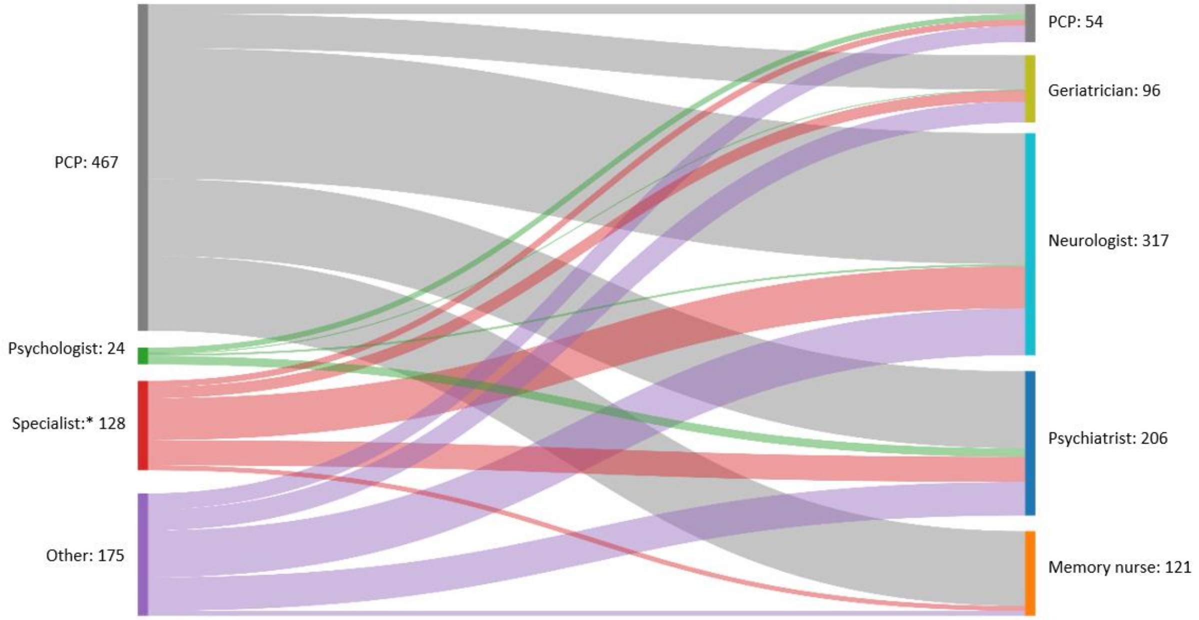

*Neurologists, psychiatrists, and geriatricians
Left side: Specialty referring to the study healthcare provider (referring physicians)
Right side: Specialty of the study healthcare provider (study healthcare provider referred to)

Supplementary Figure 2. Referral pattern: Flow of patients by specialty from the participating healthcare provider, all countries. A Sankey diagram is shown representing the flow of patients from the healthcare providers participating in the survey, by specialty (left side), to the healthcare providers referred to, by specialty (right side).

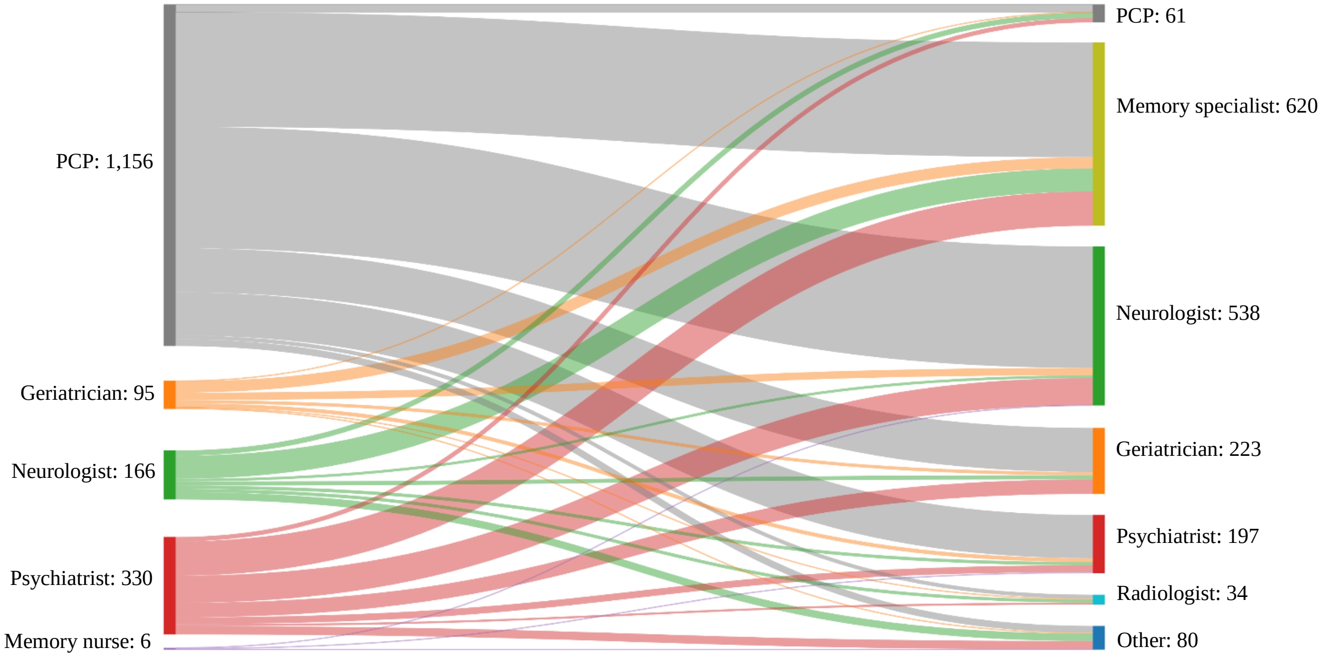

Left side: Specialty of study healthcare provider (referring healthcare provider)
Right side: Specialty of healthcare provider referred to

Supplementary Figure 3. Kaplan–Meier curve of time from first test to final diagnosis, all countries

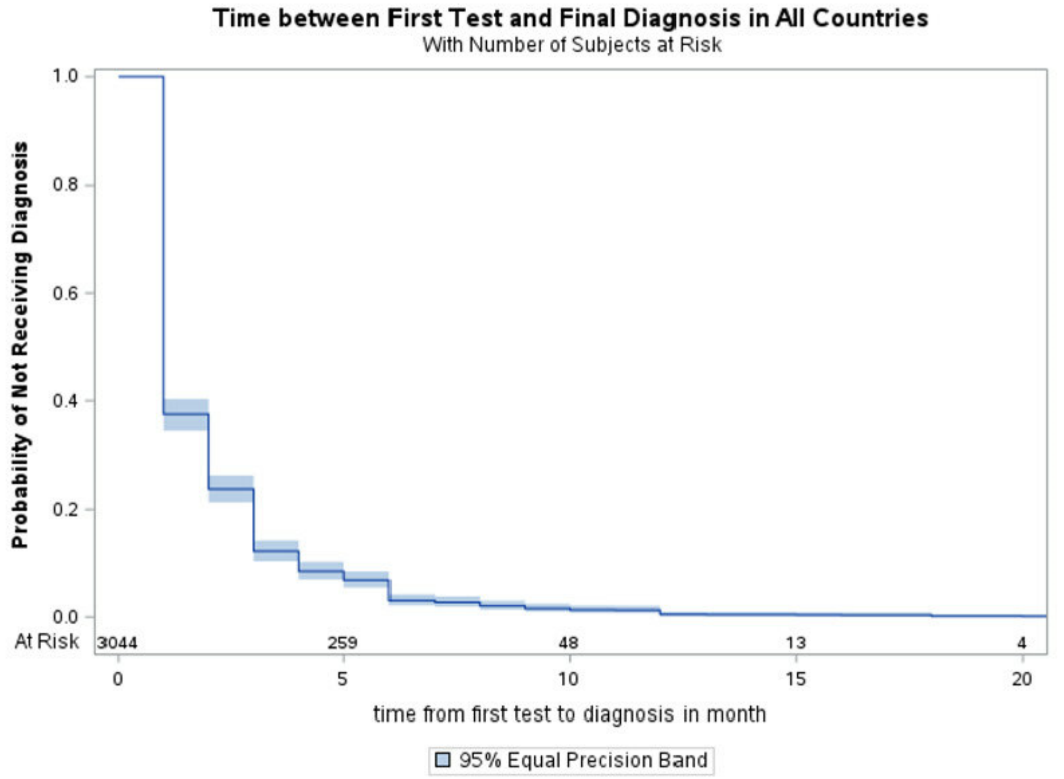

Kaplan–Meier curve of the probability of not receiving a diagnosis over time (months), for all six countries. Follow-up time refers to the time it took from patients' first presenting symptoms to receiving a final diagnosis, and is not related to the collection date of the survey data.
